# Sodium Copper Chlorophyllin Catalyzed Chemoselective Oxidation of Benzylic Alcohols and Diarylmethanes in Water

**DOI:** 10.3390/molecules23081883

**Published:** 2018-07-27

**Authors:** Shi-juan Liu, Miao Zhang, Rong Lu, Xiu-ying Li, Guang-bo Che

**Affiliations:** 1 Key Laboratory of Preparation and Application of Environmental Friendly Materials, Ministry of Education, Jilin Normal University, Changchun 130103, China; liushijuan1978@163.com (S.-j.L.); lixiuyingjilin@163.com (X.-y.L.); 2 College of Chemistry, Jilin Normal University, Siping 136000, China; 15504341392@sina.cn (M.Z.); jllr2016@163.com (R.L.)

**Keywords:** sodium copper chlorophyllin, oxidation, alcohols, chemoselectivity, water

## Abstract

We report the highly efficient and chemoselective oxidation of benzylic alcohols catalyzed by sodium copper chlorophyllin in water, producing corresponding arylcarbonyl compounds. Importantly, the catalytic system exhibits a wide substrate scope and high functional group tolerance. Moreover, secondary alcohols and even diarylmethanes were smoothly oxidized to the desired aryl ketones with excellent yields.

## 1. Introduction

The oxidation of alcohols to their corresponding carbonyls has become an essential reaction in organic chemistry [1,2]. The most important application for these carbonyl compounds is in the synthesis of fine chemicals, such as pharmaceuticals, flavors, fragrances, aniline-dyes, and food additives [3,4]. Traditionally, the oxidation of alcohols is completed using stoichiometric amounts of Cr(VI)- and Mn(VII)-based oxidants [5], which are applied in the vast majority of processes. However, costly and toxic solvents are required [6]. Copper is a low cost, abundant metal, found in various metalloproteins especially enzymes, which contribute to the binding of molecular oxygen or in selective oxidative transformations [7,8]. Cu-catalyzed selective oxidation of alcohols has received increased attention. For example, Markó et al. reported that the CuCl·Phen-catalyst can oxidize alcohols into aldehydes and ketones in the presence of diethylhydrazinodicarboxylate (DEAD-H_2_) and molecular oxygen or air [9]. Knochel et al. showed that Cu(I), in the presence of a bipyridine ligand-bearing perfluorinated ponytails, with 2, 2, 6, 6-tetramethylpiperidinyl-1-oxy (TEMPO) and oxygen, can mediate the fluorous biphasic oxidation of primary, secondary, allylic, and benzylic alcohols [10]. Similarly, Gree et al. developed a series of [Cu-TEMPO]-mediated oxidation reactions of primary and/or secondary alcohols [11,12,13,14,15]. Birinchi et al. described a homogeneous catalyst based on the polymeric coordination complex [CuCl_2_(4-CNpy)_2_]n (1; 4-CNpy = 4-cyanopyridine) that catalyses the oxidation of both primary and secondary alcohols using TBHP(aq.) as the oxidant [16]. In addition, the selective aerobic oxidation of alcohols into their corresponding aldehydes or ketones was also possible using a two-component system, VO(acac)_2_/DABCO in an ionic liquid ([bmim]PF_6_) [17]. From both economic and environmental points of view, the quest for efficient catalytic systems that use simple, effective, environmentally friendly, and inexpensive catalysts for the transformation of alcohols into carbonyl compounds on an industrial scale remains a challenge.

**Figure 1:**
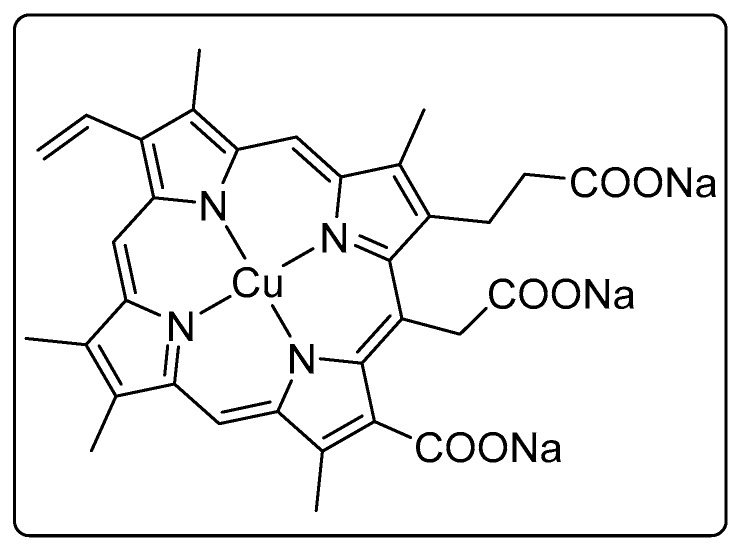
The structure of sodium copper chlorophyllin (SCC; C_34_H_31_CuN_4_Na_3_O_6_).

Chlorophyll, a highly abundant tetrapyrollic compound, is the essential pigment necessary for photosynthesis to occur in plants, algae, and cyanobacteria. Sodium copper chlorophyllin (C_34_H_31_CuN_4_Na_3_O_6_, SCC; Figure 1) is a semi-synthetic, water-soluble derivative of chlorophyll and is widely used in the food and medicine industries [18,19,20]. Due to its characteristic tetrapyrrole structure, the photochemical properties of SCC have been well studied by organic photovoltaics and optical spectroscopy [21,22,23,24,25]. However, the effectiveness of SCC as a catalyst in organic reactions has yet to be explored in-depth [26]. Herein, we describe a highly efficient and practical protocol for the selective catalytic oxidation of benzylic alcohols to aldehydes and carboxylic acids using SCC as a green, safe, and cheap metal-copper catalyst in water.

## 2. Results and Discussion

Optimization of the Reaction Conditions

The optimization of our oxidation reaction was completed using benzyl alcohol (1 mmol) and TBHP (tert-Butyl hydroperoxide), as the oxidant, in the presence of SCC, as the catalyst, and 4-methylpyridine, as an additive, in water (Table 1). After 10 h at 30 °C, benzaldehyde **2a** and benzoic acid **3a** were obtained in 46% and 15% yields, respectively (entry 1, Table 1). By increasing the reaction temperature to 60 °C, the reaction favored oxidized product **2a** (entry 2, Table 1). In the absence of SCC, the desired product was only obtained with a 18% yield (entry 3, Table 1). Further increase of the reaction temperature to 70 °C or the SCC catalyst to 2 mmol % did not significantly affect the chemoselectivity and conversion (entries 4–6, Table 1). Reducing the reaction time to 5 h, although highly selective for the oxidative product, only provided a 43% yield of **2a** (entry 7, Table 1). Notably, an increase in the amount of TBHP had a noticeable effect on the chemoselectivity of the reaction (entries 8–10, Table 1). Moreover, when the reaction was completed in the presence of 3.0 equiv. of TBHP at 80 °C, benzoic acid **3a** was obtained with a 95% yield with high chemoselectivity (entry 11, Table 1).

Several benzyl alcohols underwent SCC-catalyzed oxidation using 1 equiv. of TBHP, leading to, in most cases, the chemoselective formation of aromatic aldehyde products **2b**–**h** (Table 2). A variety of substituted benzyl alcohols were examined, with the more electron-rich substrates producing good yields with high chemoselectivity (**2b** and **2g**, entries 1 and 6, Table 2). However, benzyl alcohols bearing electron-poor substituents produced desired aldehydes **2c**–**f** and **2h** (entries 2–5, and 7, respectively, Table 2) in somewhat diminished yields (14–56%).

Furthermore, we evaluated the SCC catalyst for the oxidation of benzyl alcohols in the presence of TBHP (3 equiv.) at 80 °C (Table 3). Both electron-rich and -poor substituted benzyl alcohols were tolerated, affording arylcarboxylic acids **3b**–**j** with excellent chemoselectivity and high yield (entries 2–10, Table 3). Despite the steric congestion, the reactions proceeded to completion within 15 h.

To explore the generality of our protocol, a variety of secondary alcohols were investigated. The results are summarized in Table 4. Substituted 9*H*-fluoren-9-ols were successfully oxidized in the presence of 1 mmol % SCC, 70% TBHP (3 equiv.), and acetone/H_2_O (1 mL/1 mL) as a solvent, to produce 9*H*-fluoren-9-one products **3k** and **3l** with excellent yields. Similarly, the oxidation of diphenylmethanol derivatives were also performed under the same conditions and produced the desired products **3m**–**p** in 81–98% yields. In addition, the oxidation of 1-(naphthalen-2-yl) ethan-1-ol was also possible, producing 1-(naphthalen-2-yl) ethan-1-one **3q** with a 66% yield (Supplementary materials).

We next examined if the oxidation of diarylmethane would enable the synthesis of diaryl ketones (Table 5). Gratifyingly, diarylmethane substrates bearing 2-Br, 2.7-di-*tert*-butyl substituted 9*H*-fluorenes or diphenylmethane substituents were smoothly oxidized to produce desired products, **3l**, **3r**, and **3m**, with excellent yields (Supplementary materials)..

**Scheme 1:**
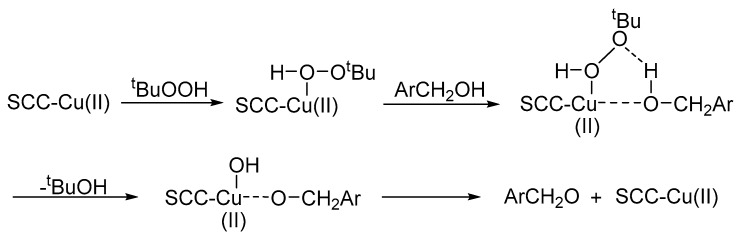
Possible mechanistic pathway for smoothly sodium copper chlorophyllin (SCC)-catalyzed oxidation of benzyl alcohols.

Based on the above results, a possible mechanistic pathway for the oxidation of benzyl alcohol with TBHP over SCC is proposed in Scheme 1. Under the reaction conditions, SCC, TBHP, and benzyl alcohol initially form pentacyclic transition-state inter-mediates, and then dehydrate to produce benzaldehyde. The addition of 4-methylpyridine as a base promoter accelerates the oxidation of alcohols by rapid decomposition of the catalyst-substrate intermediate that forms during the reaction under basic conditions [27,28] (Supplementary materials).

## 3. Materials and Methods

Chemicals were obtained commercially and used as received. Nuclear magnetic resonance (NMR) spectra were recorded on a Bruker DPX–400 spectrometer (Bruker Co., Billerica, MA, USA) using tetramethylsilane (TMS) as the internal standard. Electric impact ionization (EI) –Mass spectrum was measured on a gas chromatography time of flight high resolution mass spectrometry (GCTOF-HRMS) (Waters Co, Milford, MA, USA) or GC-MS (Agilent 7890A/5975C, Santa Clara, CA, USA) instrument. All products were isolated via short chromatography on a silica gel (200–300 mesh) column using petroleum ether (60–90 °C), unless otherwise noted. Alcohols and diarylmethanes were of analytical grade quality, purchased from Adamas-beta Pharmaceuticals, Inc. (Shanghai, China) Compounds described in the literature were characterized by ^1^H-NMR spectra compared to reported data.

### 3.1. General Procedure for the Selective Oxidation of Benzyl Alcohols Catalyzed by SCC

A solution of benzyl alcohol (1 mmol), SCC (1 mol %), 70% TBHP (1 mmol or 3 mmol), and 4-methylpyridine (1.0 mmol) in H_2_O (2 mL) was stirred at 60 °C (or 80 °C) for 10 h (or 15 h). The reaction mixture was quenched with a saturated solution of sodium thiosulfate (5 mL) and extracted using dichloromethane (3 × 10 mL). The combined organic layers were dried over anhydrous Na_2_SO_4_, filtrated, and then the solvent was removed under reduced pressure. The residue was purified by flash column chromatography on silica gel with petroleum ether/ethyl acetate as the eluent to obtain the desired product.

### 3.2. General Procedure for the Oxidation of Secondary Alcohols (or Diarylmethanes) Catalyzed by SCC

A solution of secondary alcohol (or diarylmethane) (0.5 mmol), SCC (1 mol %), 70% TBHP (1.5 mmol), and 4-methylpyridine (1.5 mmol) in acetone/H_2_O (1:0.5 mL) was stirred at 80 °C for 10 h. The reaction mixture was quenched with the saturated solution of sodium thiosulfate (5 mL) and extracted with dichloromethane (3 × 10 mL). The combined dichloromethane extracts were dried over anhydrous Na_2_SO_4_, filtrated, and then the solvent was removed under reduced pressure. The residue was purified by flash column chromatography on silica gel with PE/EtOAc as the eluent to obtain the desired products.

## 4. Conclusions

In this work, we showed that the SCC-catalyzed oxidation of benzyl alcohols in water is an efficient and highly chemoselective method to construct arylformaldehydes and arylformic acids. Our methodology is also suitable for the efficient oxidation of secondary alcohols and diarylmethane derivatives to produce the corresponding ketones. Further studies to explore the intriguing catalytic abilities of the SCC system are currently underway in our laboratory.

## Figures and Tables

**Table 1:** The optimization of the oxidation reaction ^a^.

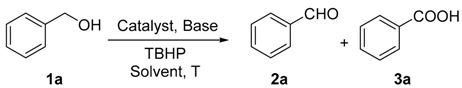						
Entry	TBHP (Equiv.)	SCC (%)	T (°C)	T (h)	Yield (%) ^b^	
					2a	3a
1	1	1	30	10	46	15
2	1	1	60	10	82	17
3	1	0	60	10	18	-
4	1	1	70	10	81	16
5	1	2	70	10	85	14
6	1	1	80	10	74	17
7	1	1	80	5	43	5
8	1.5	1	60	10	60	38
9	2.0	1	60	10	30	61
10	3.0	1	60	10	18	75
11	3.0	1	80	10	-	95

^a^ Reaction conditions: benzyl alcohol (1 mmol), sodium copper chlorophyllin (SCC; C_34_H_31_CuN_4_Na_3_O_6_; 1 mol %), 4-methylpyridine (1 mmol), H_2_O (2 mL). ^b^ Isolated yield.

**Table 2:** The oxidation of benzyl alcohol catalyzed by sodium copper chlorophyllin (SCC) ^a^.

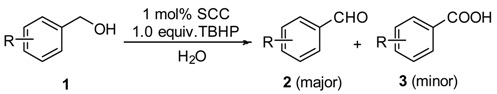					
Entry	R/1	T (°C)	t (h)	Yield (%) ^b^	
				2	3
1	4-Me/**1b**	60	10	**2b**/75	-
2	4-Br/**1c**	80	10	**2c**/52 ^c^	-
3	4-NO_2_/**1d**	60	10	**2d**/45	**3d**/18%
4	3-F/**1e**	60	12	**2e**/56	**3e**/35%
5	3-F, 4-OMe/**1f**	60	10	**2f**/14	-
6	2-Me/**1g**	60	10	**2g**/60	-
7	2-F/**1h**	60	10	**2h**/33	-

^a^ Reaction conditions: benzyl alcohol (1 mmol), SCC (1 mol %), TBHP (1 mmol), H_2_O (2 mL), 80 °C, 10 h, 4-methylpyridine (1 mmol). ^b^ Isolated yield, ^c^ TBHP (2 mmol).

**Table 3:** The oxidation of benzyl alcohol catalyzed by SCC ^a^.

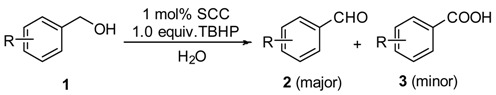				
Entry	R/1	t (h)	Yield (%) ^b^	
			2	3
1	H/**1a**	10	-	**3a**/95
2	4-Me/**1b**	15	-	**3b**/97
3	4-Br/**1c**	15	-	**3c**/77
4	4-NO_2_/**1d**	15	**2d**/12	**3d**/88
5	3-F/**1e**	15	-	**3e**/93
6	3-F, 4-OMe/**1f**	15	-	**3f**/95
7	2-Me/**1g**	15	-	**3g**/97
8	2-F/**1h**	15	**2h**/5	**3h**/91
9	3-MeO/**1i**	15	-	**3i**/93
10	2-Cl/**1j**	15	-	**3j**/89

^a^ Reaction conditions: benzyl alcohol (1 mmol), SCC (1 mol%), H_2_O (2 mL) as solvent, TBHP (3 equiv.), 4-methylpyridine (1 mmol), 80 °C. ^b^ Isolated yield.

**Table 4:** The oxidation of secondary alcohols catalyzed by SCC ^a^.

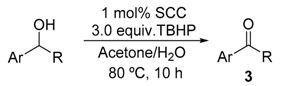			
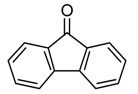 **3k**/98%	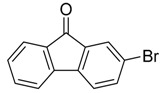 **3l**/98%	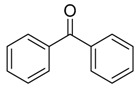 **3m**/96%	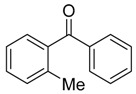 **3n**/81%
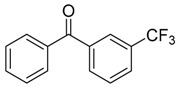 **3o**/98%	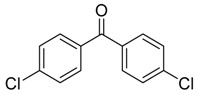 **3p**/98%	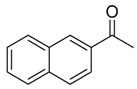 **3q**/66%	

^a^ Reaction conditions: 0.5 mmol secondary alcohol, SCC (1 mol%), TBHP (3.0 equiv.), 4-methylpyridine (1.0 equiv.), acetone/H_2_O = 1 mL/1 mL, 80 °C, 10 h.

**Table 5:** The oxidation of diarylmethanes catalyzed by SCC ^a^.

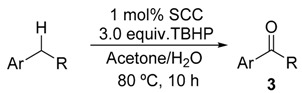		
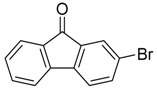 **3l**/97%	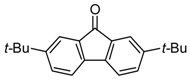 **3r**/97%	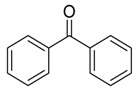 **3m**/95%

^a^ Reaction conditions: 0.5 mmol diarylmethane, SCC (1 mol %), TBHP (3.0 equiv.), 4-methylpyridine (1.0 equiv.), acetone/H_2_O = 1 mL/1 mL, 80 °C, 10 h.

## Supplementary Materials

Supplementary materials are available online, the charts of ^1^H-NMR of products.
