# Supplementary material for: Sodium Copper Chlorophyllin Catalyzed Chemoselective Oxidation of Benzylic Alcohols and Diarylmethanes in Water
Source: Molecules. 2018 Jul 27;23(8):1883. doi: 10.3390/molecules23081883 (PMC6222594; doi:10.3390/molecules23081883)

Supporting Information for

Sodium copper chlorophyllin catalyzed chemoselective oxidation of alcohol and diarylmethanes in water

Shi-juan Liu[a,b], Miao Zhang[b], Rong Lu[b], Xiu-ying Li[a,b], Guang-bo Che[a,*]

[a] Key Laboratory of Preparation and Application of Environmental Friendly Materials (Jilin Normal University), Ministry of Education, Changchun, 130103, China. liushijuan1978@163.com (S.-j. L.); 15504341392@sina.cn (M.Z.); jllr2016@163.com (R. L.); lixiuyingjilin@163.com (L.-x. L.)

[b] College of Chemistry, Jilin Normal University, Siping, 136000, China. guangboche@jlnu.edu.cn (G. Che).

* Correspondence: e-mail: guangboche@jlnu.edu.cn (G. Che).

Copies of ^{1}H NMR……………………………………………S2–S14

4-methylbenzaldehyde (2b)

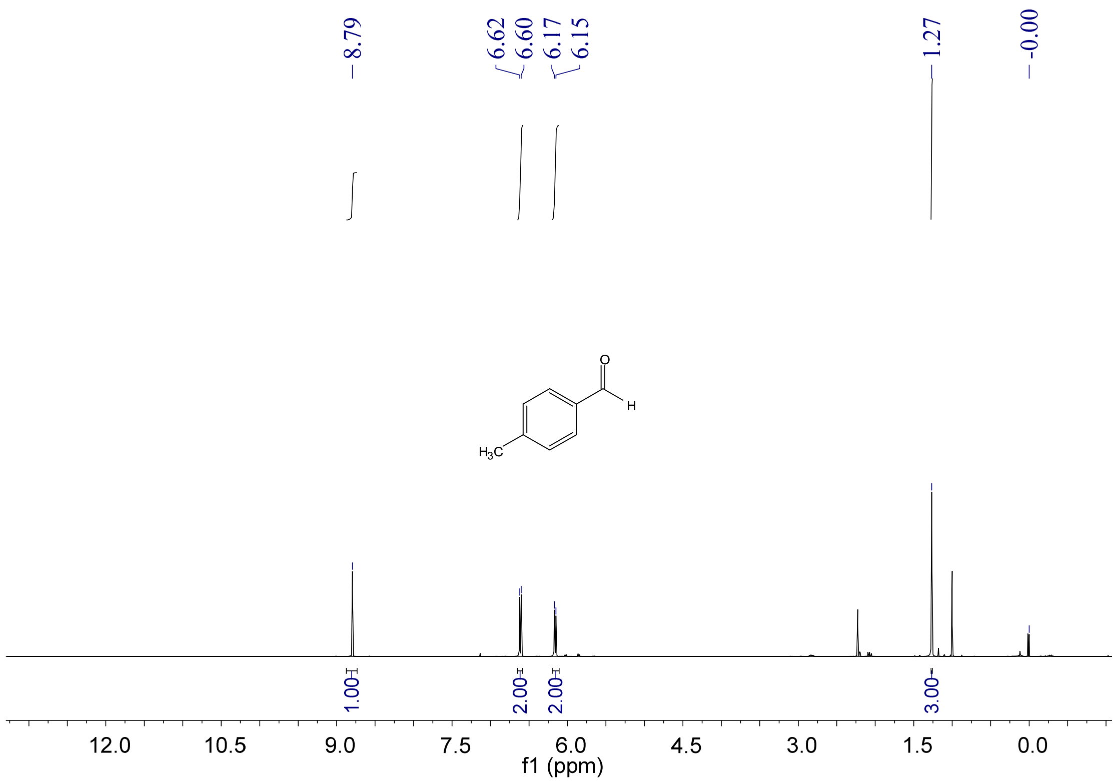

4-bromobenzaldehyde (2c)

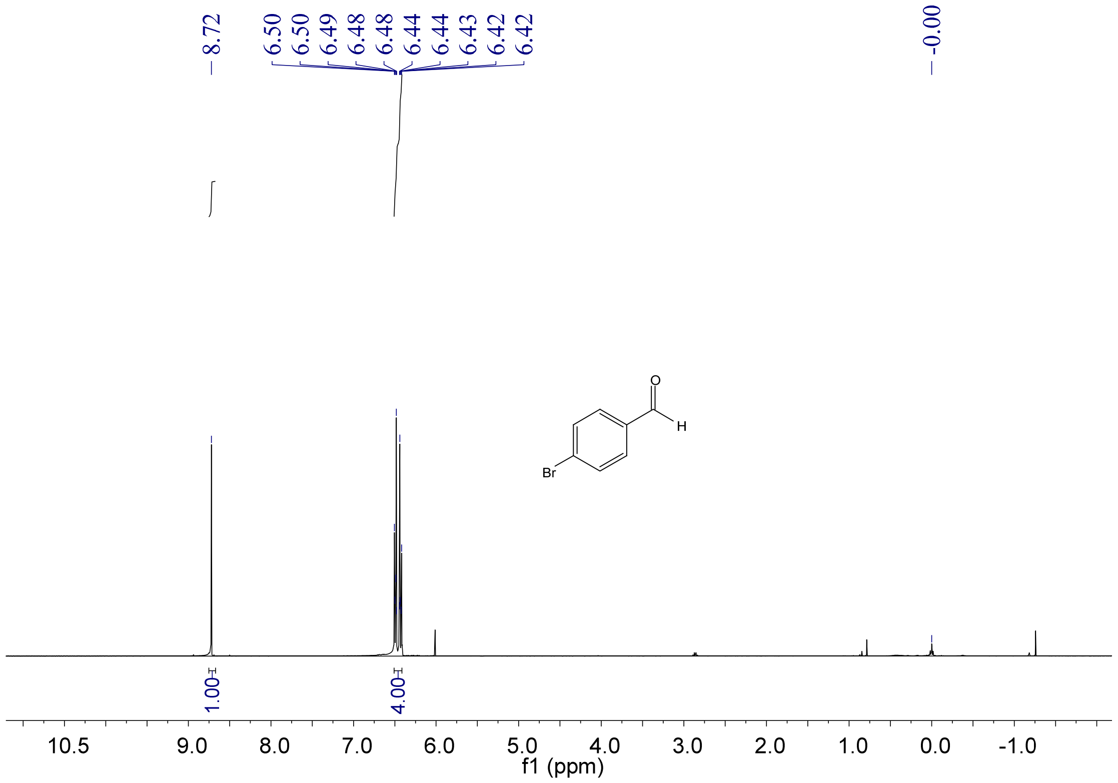

4-nitrobenzaldehyde (2d)

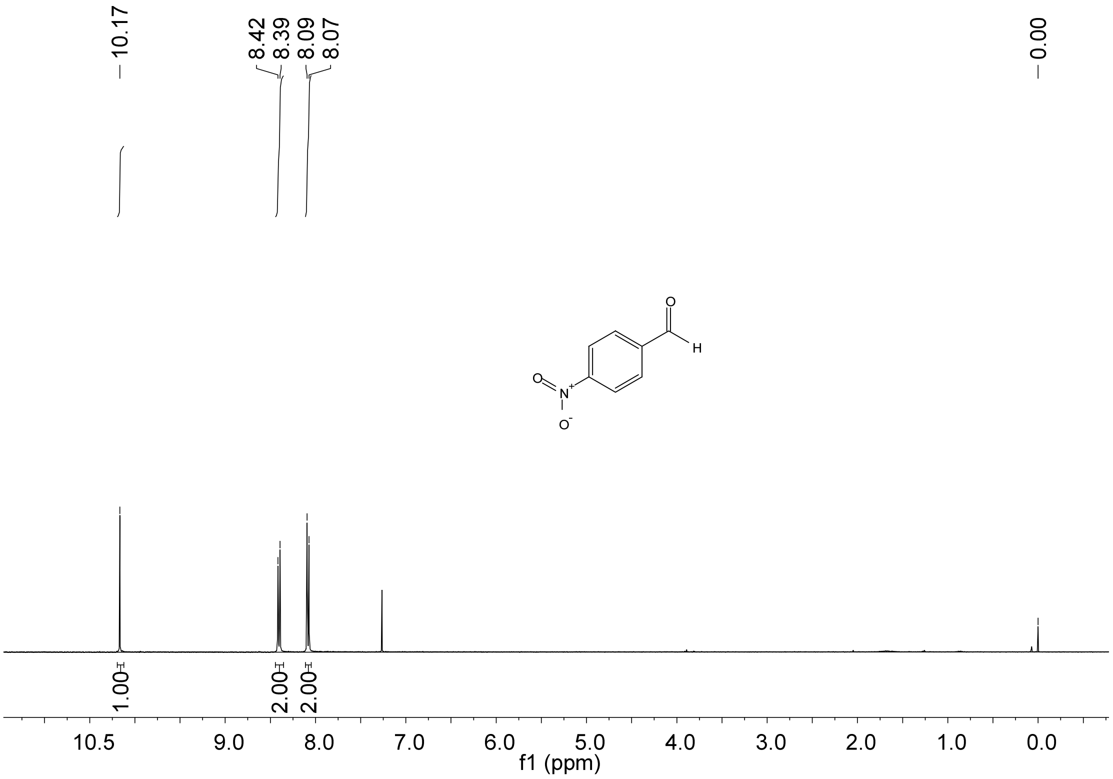

3-fluorobenzaldehyde (2e)

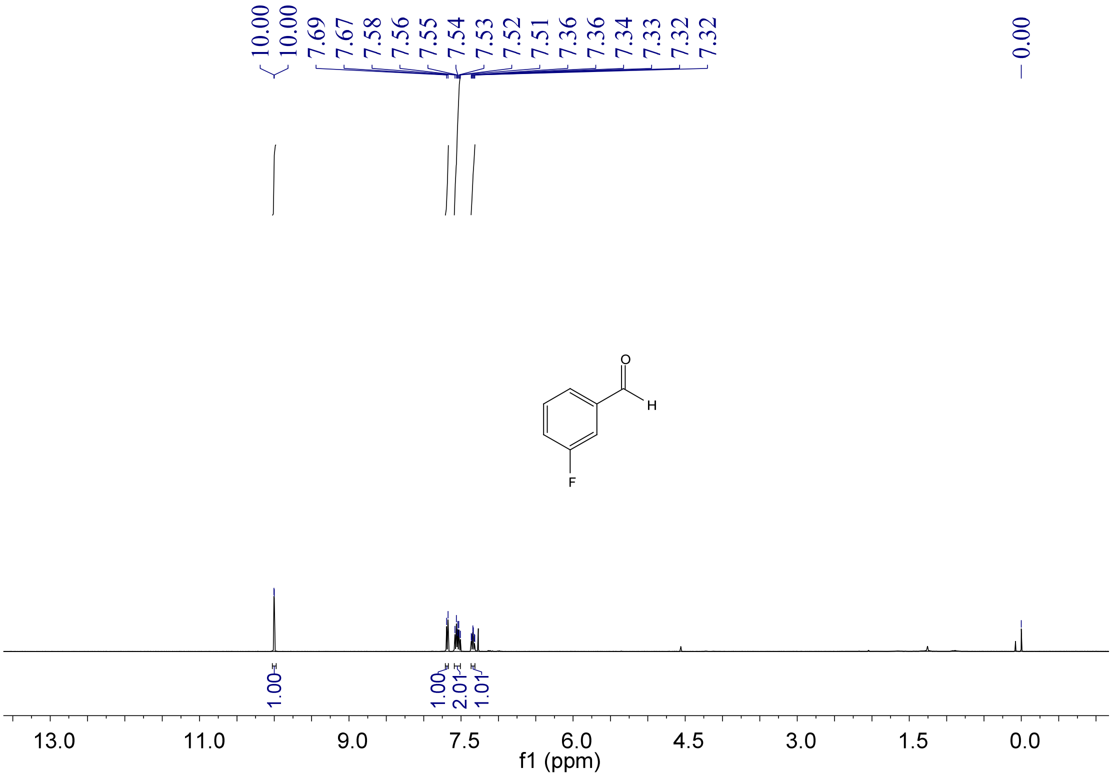

3-fluoro-4-methoxybenzaldehyde (2f)

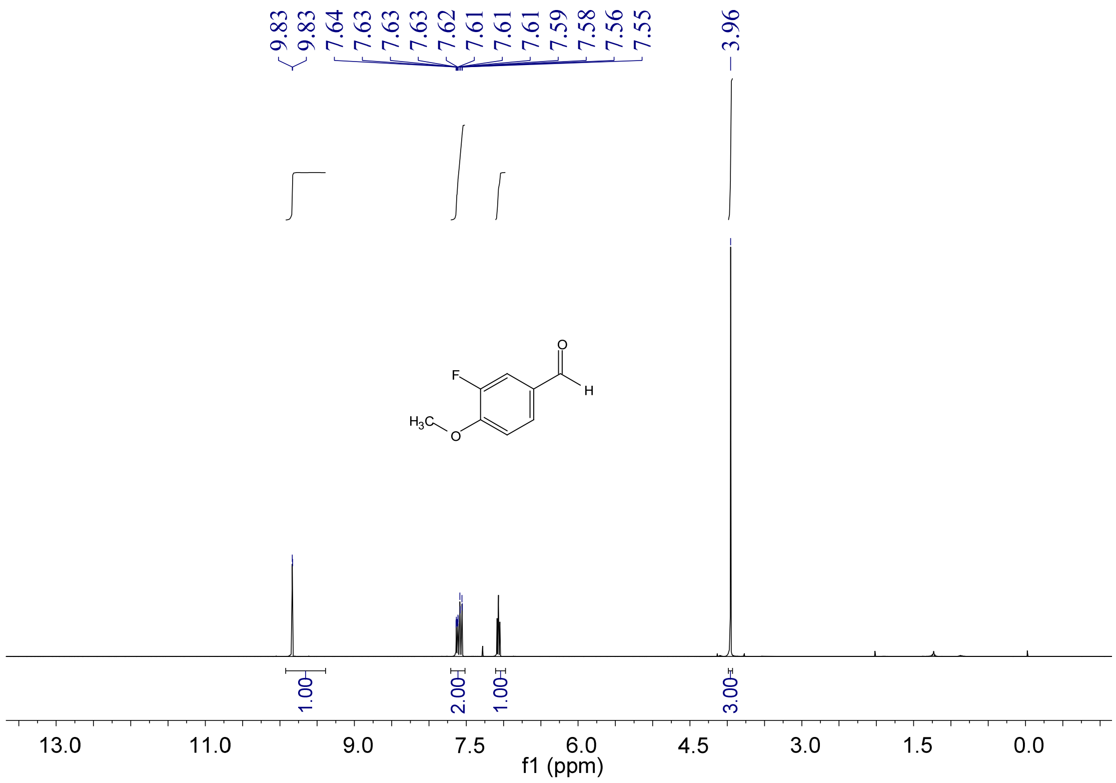

2-methylbenzaldehyde (2g)

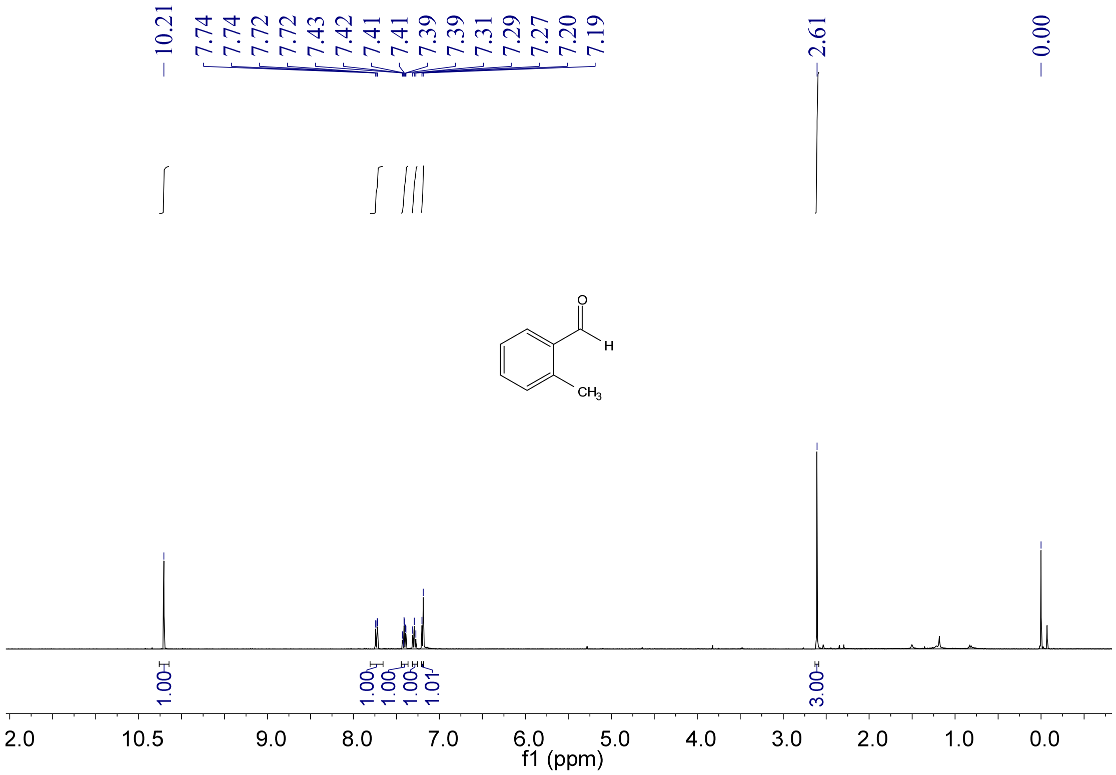

2-fluorobenzaldehyde (2h)

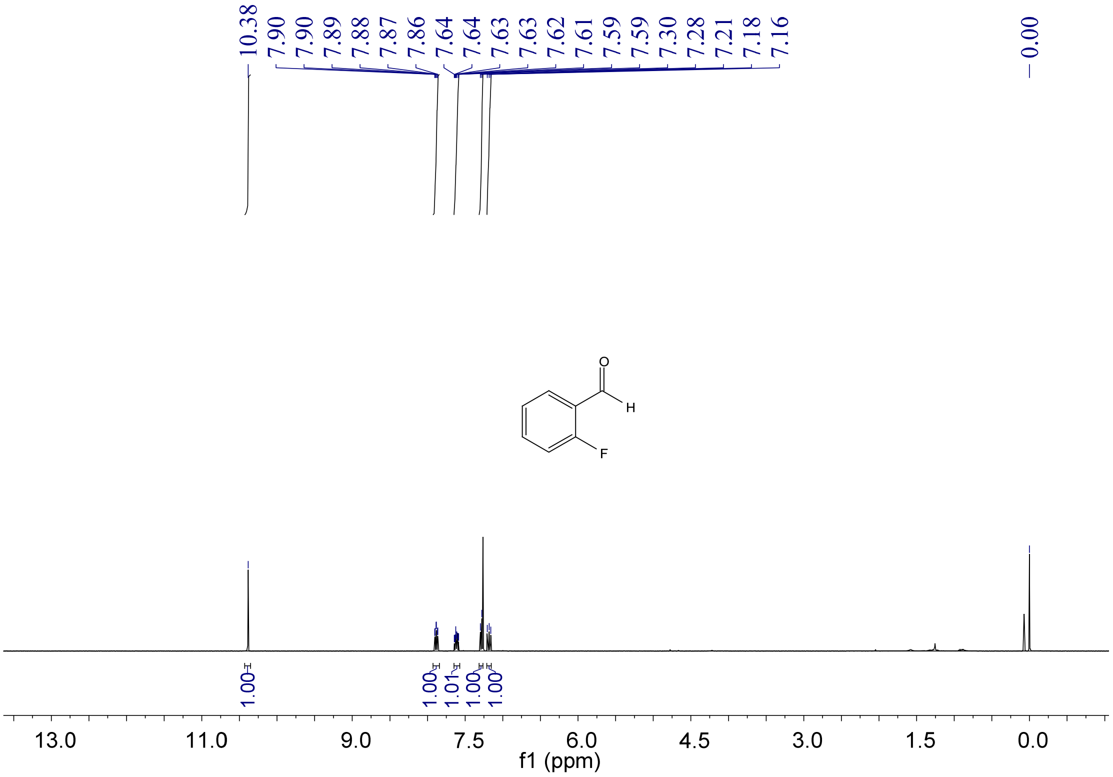

benzoic acidc (3a)

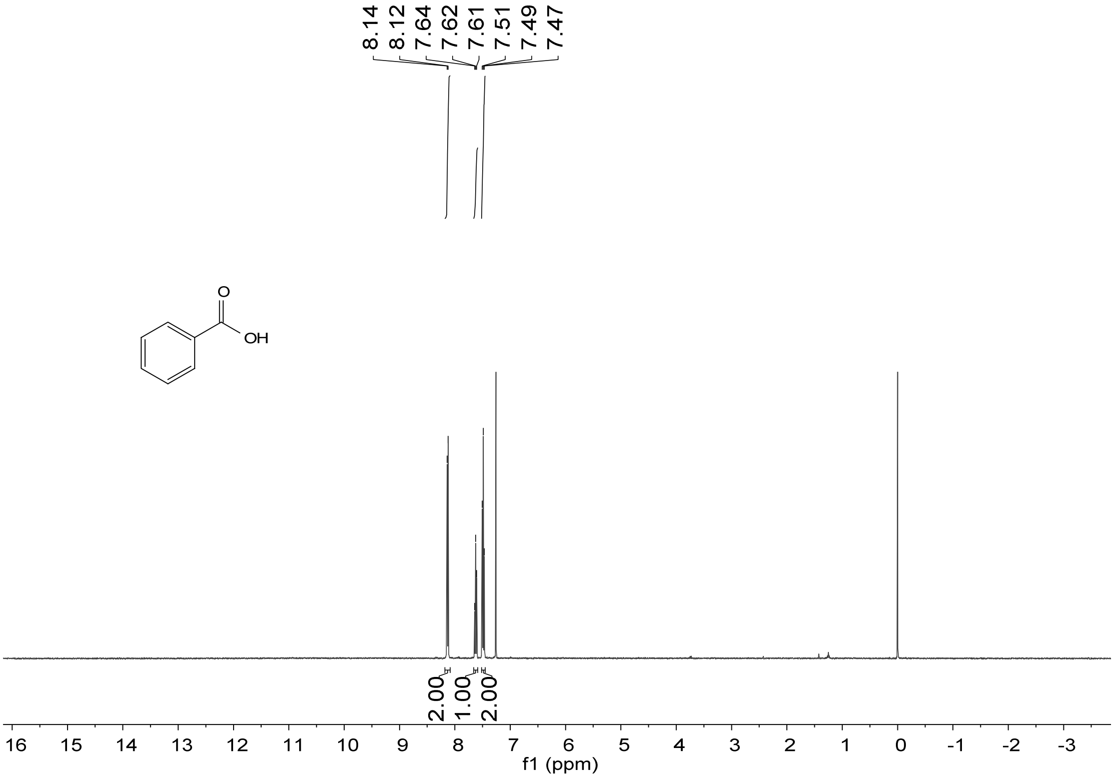

4-methylbenzoic acid (3b)

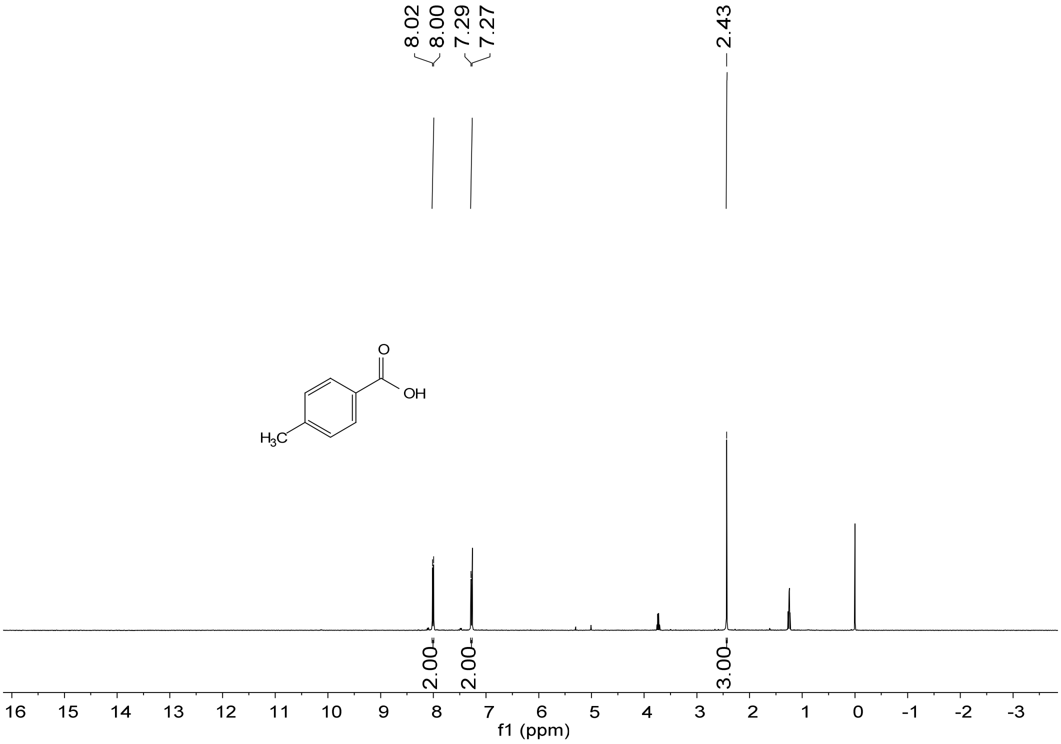

4-bromobenzoic acid (3c)

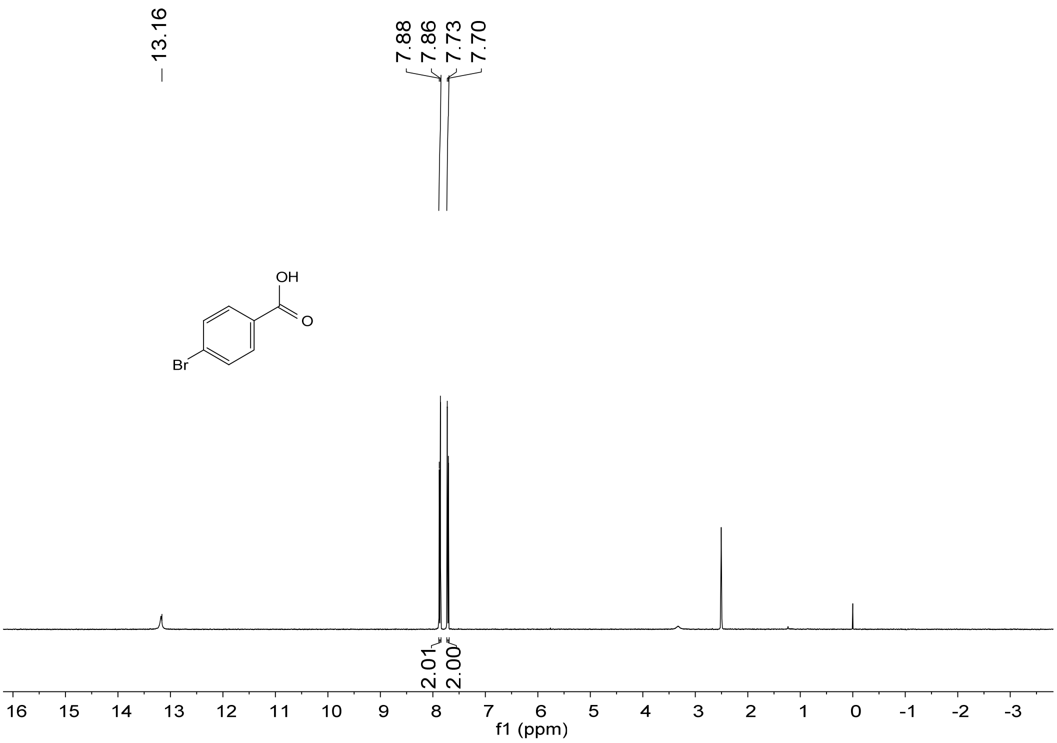

4-nitrobenzoic acid (3d)

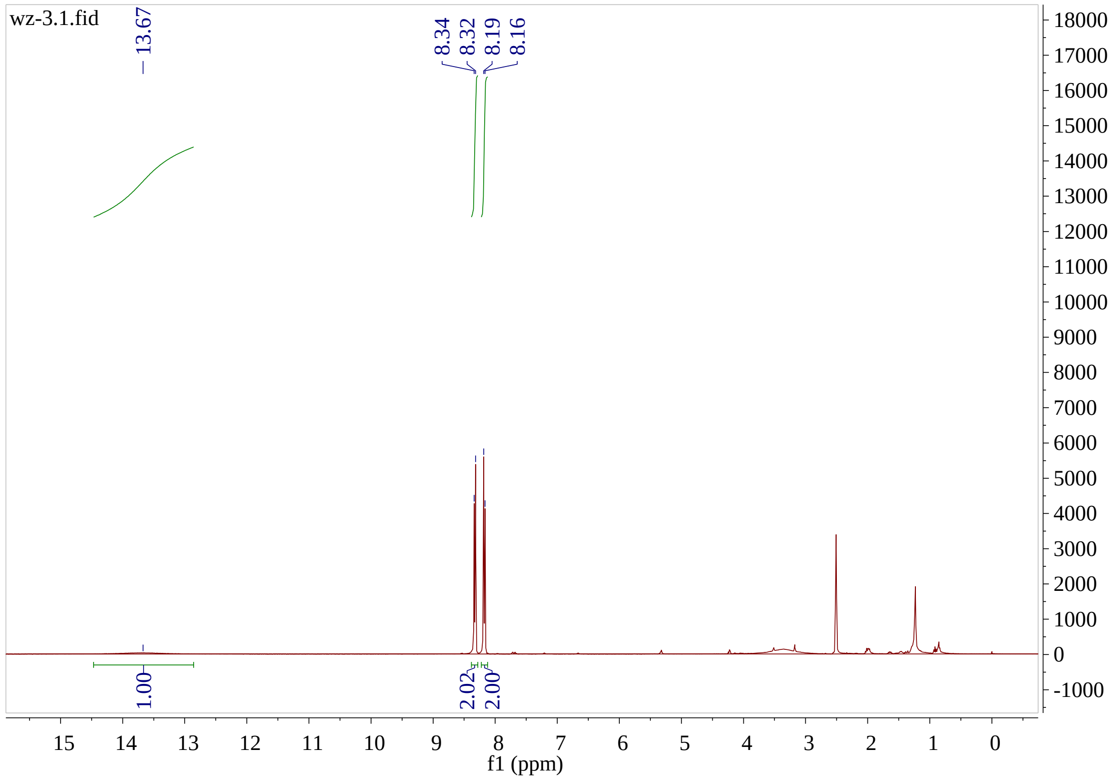

3-fluorobenzoic acid (3e)

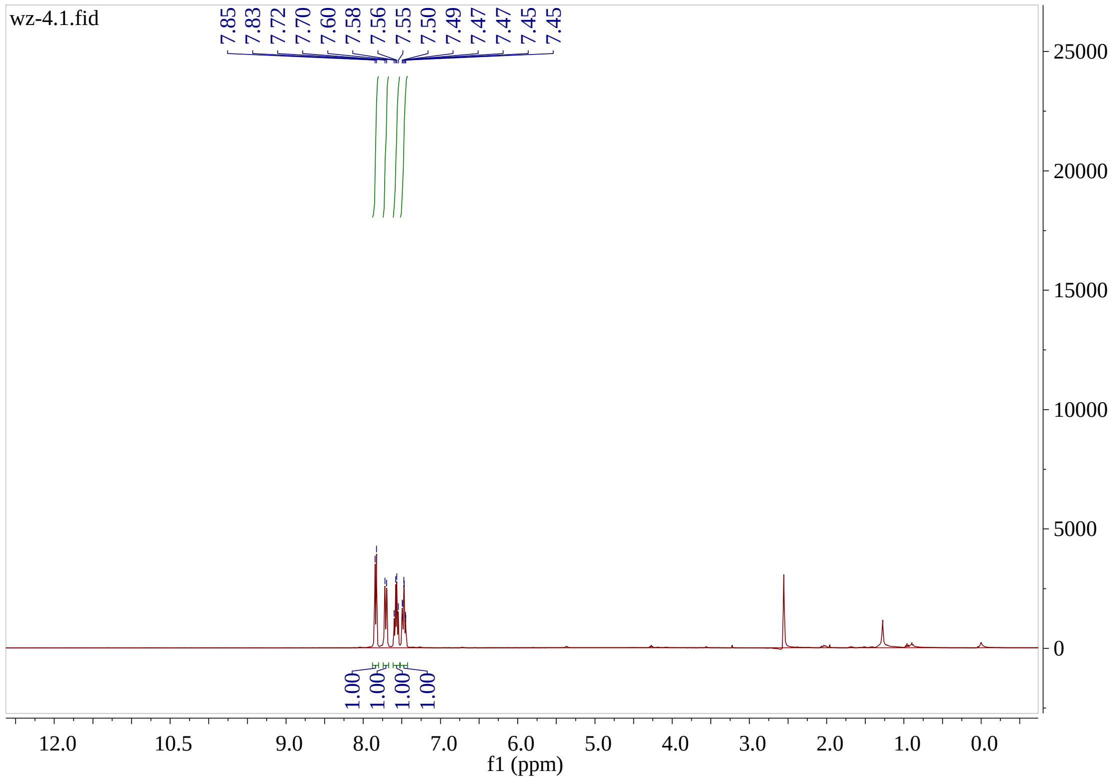

3-fluoro-4-methoxybenzoic acid (3f)

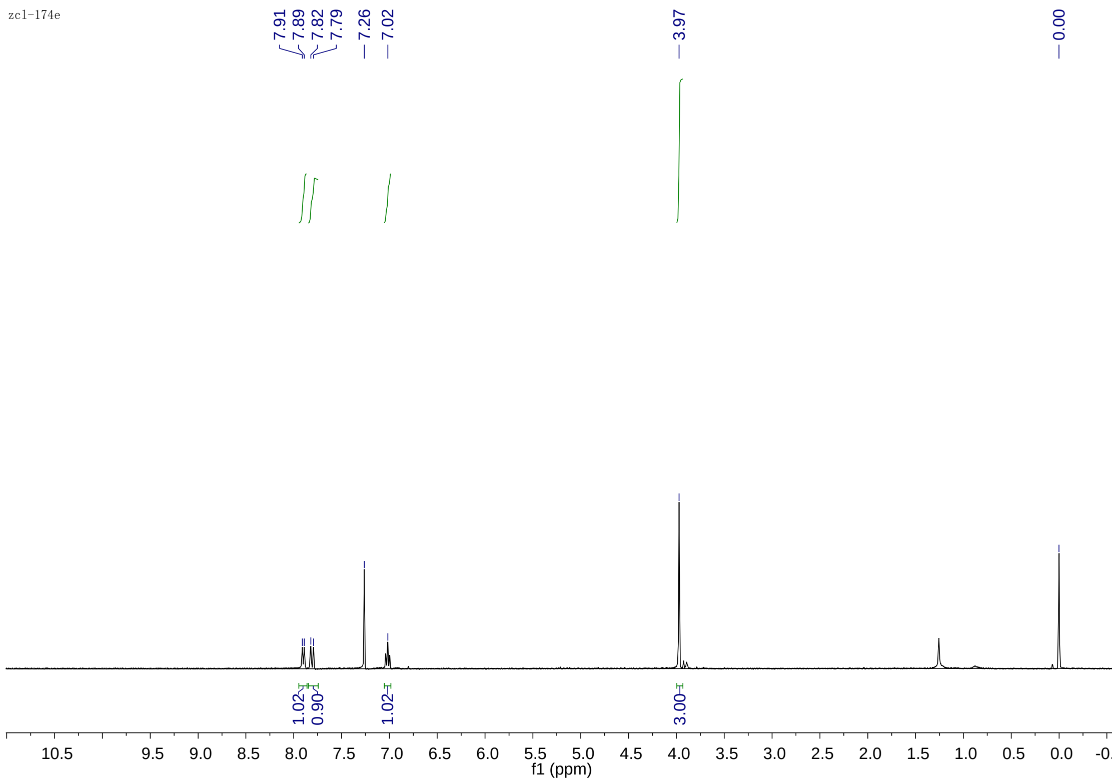

3-methoxybenzoic acid (3i)

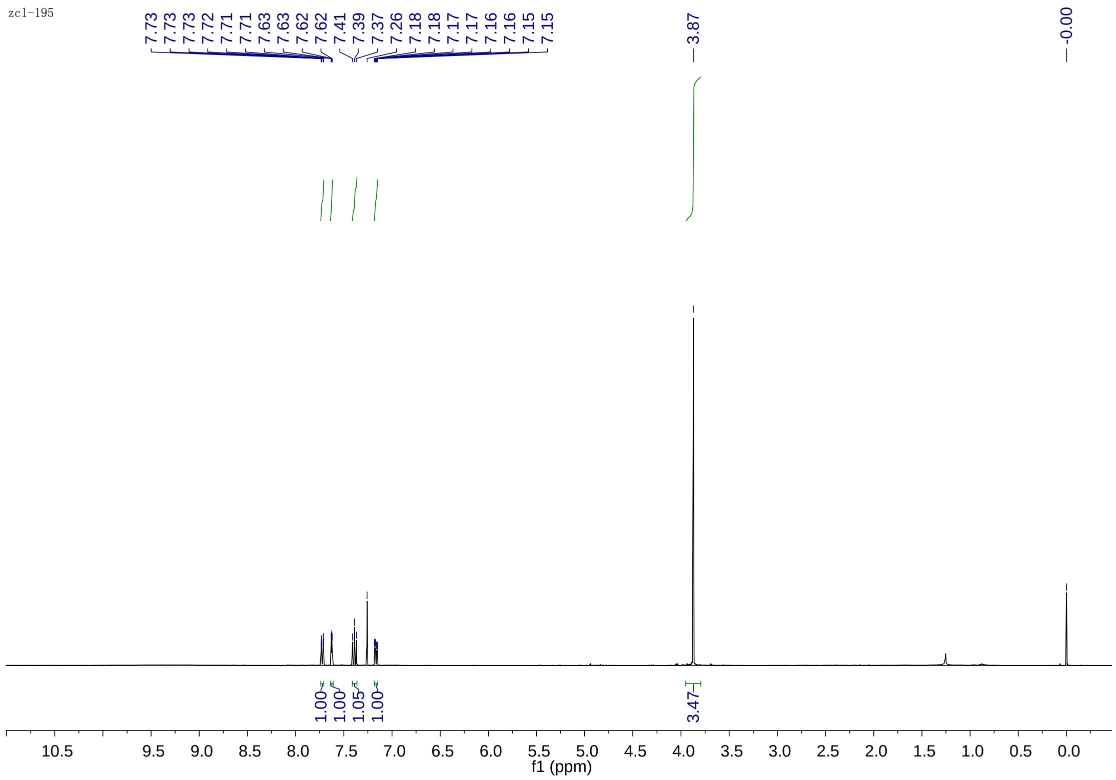

2-methylbenzoic acid (3g)

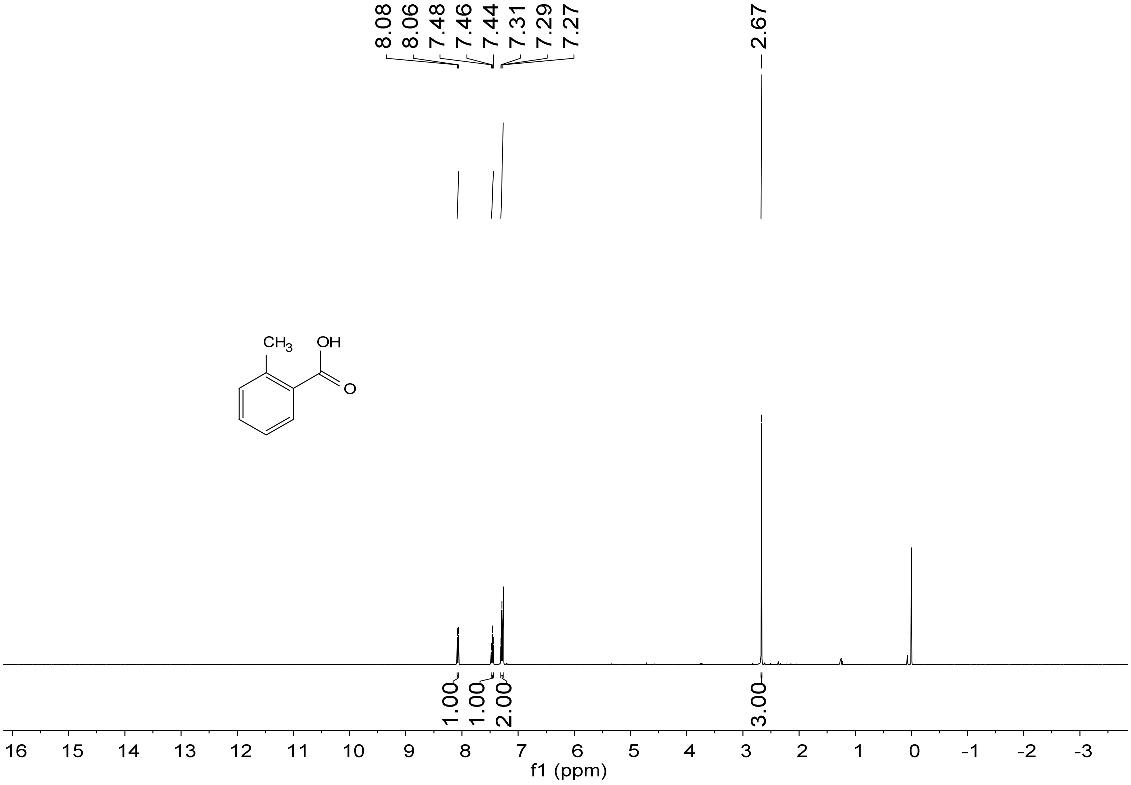

2-fluorobenzoic acid (3h)

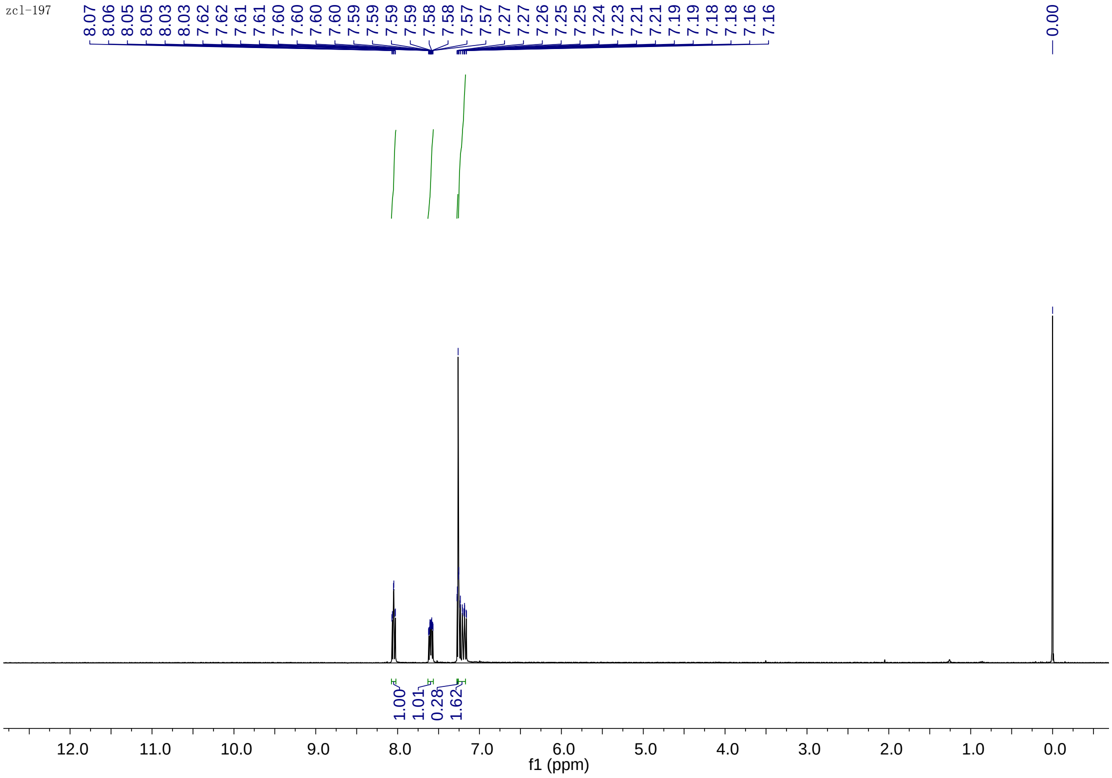

2-chlorobenzoic acid (3j)

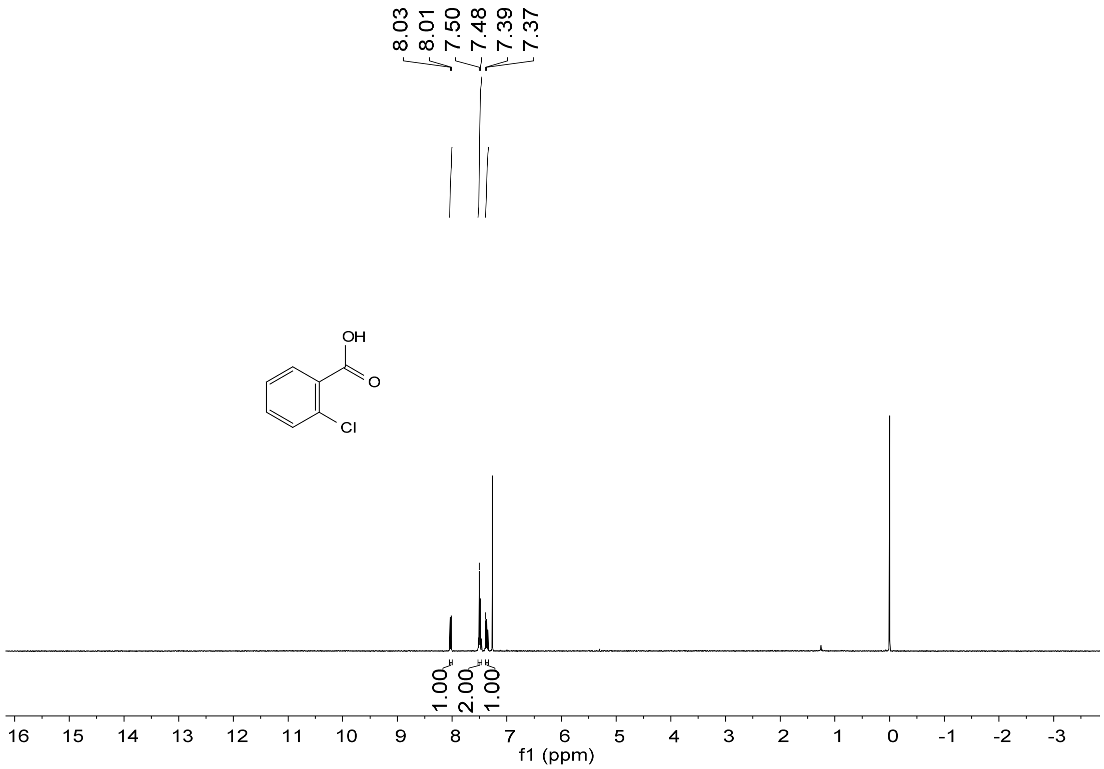

9H-fluoren-9-one (3k)

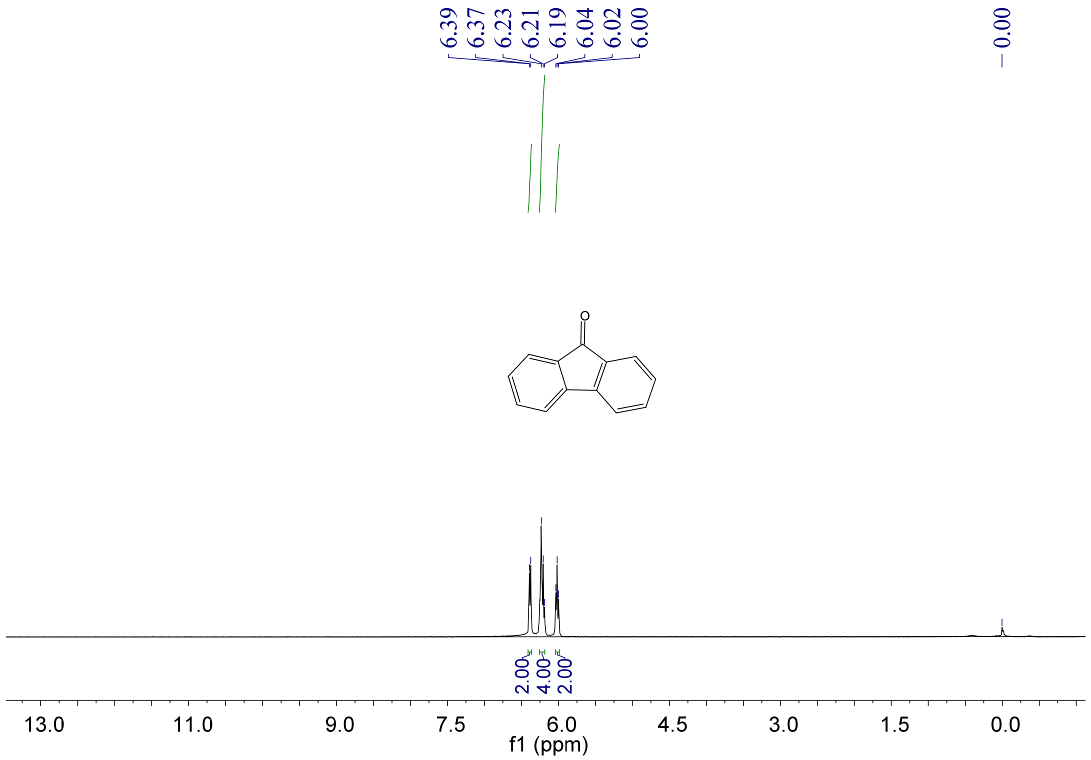

2-bromo-9H-fluoren-9-one (3l)

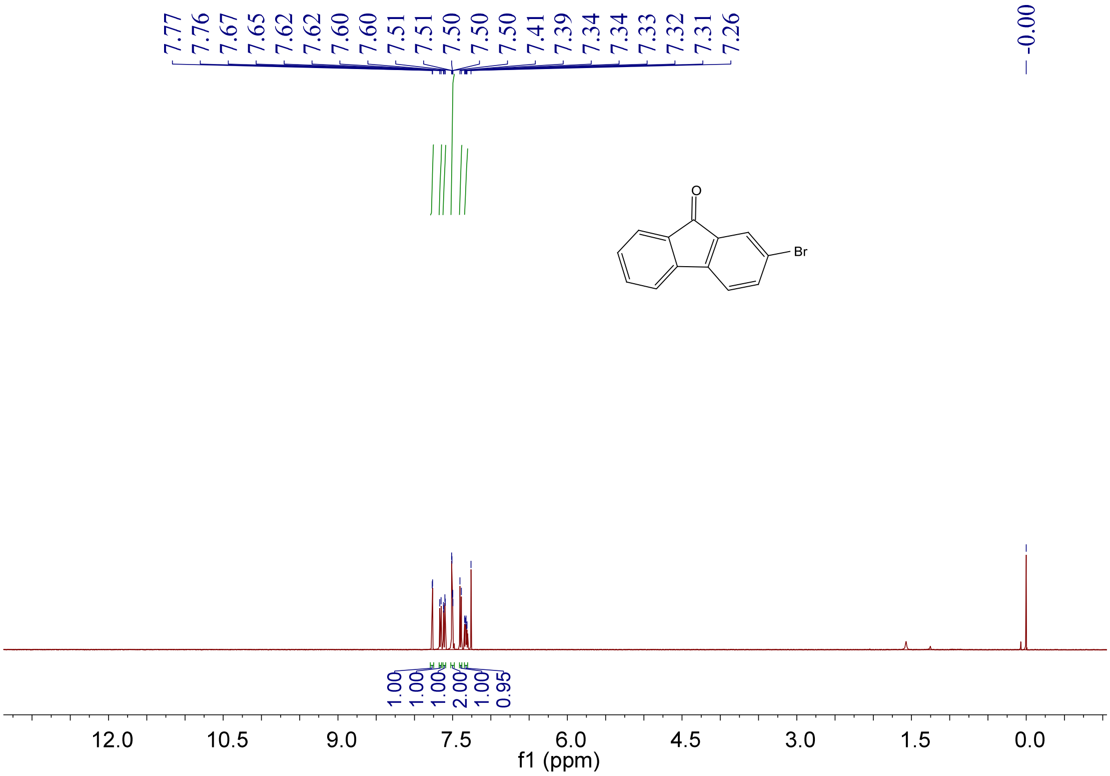

benzophenone (3m)

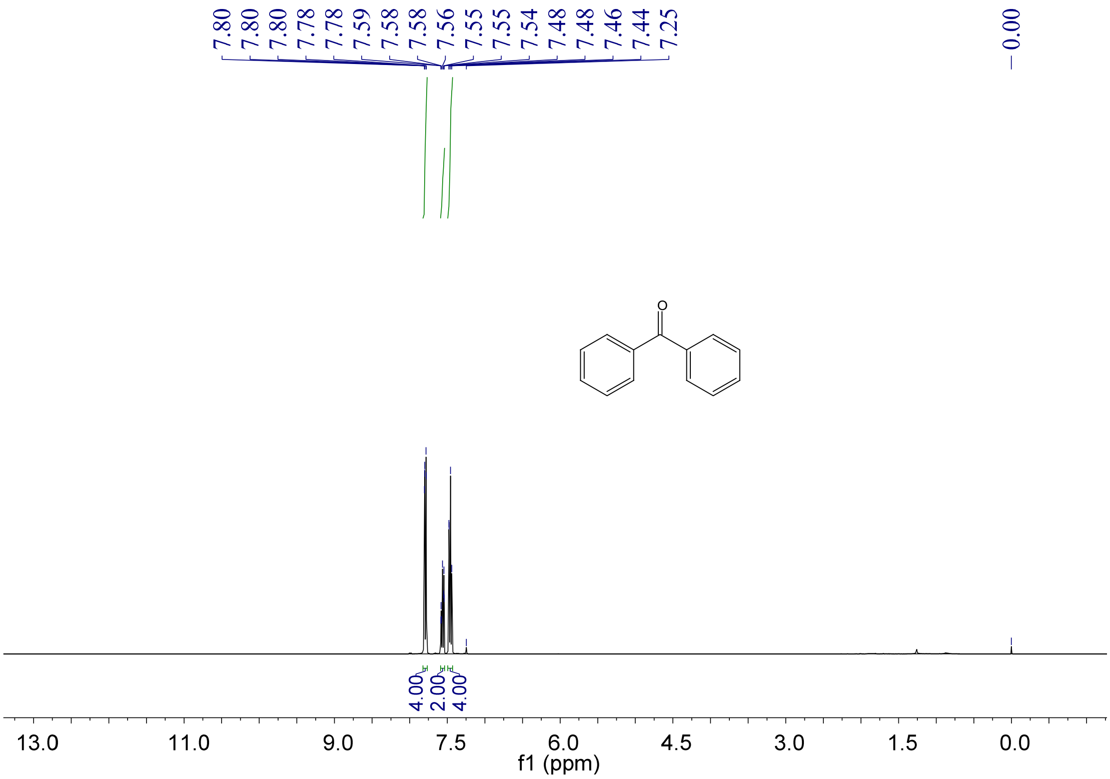

phenyl(o-tolyl)methanone (3n)

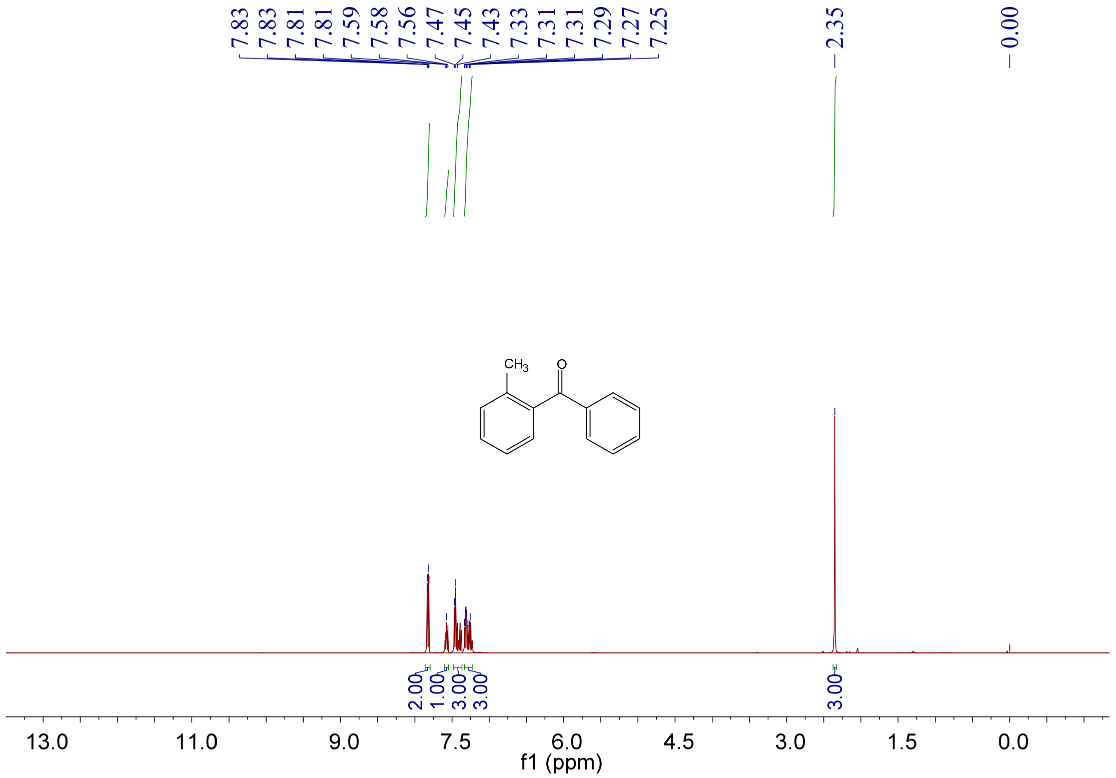

phenyl(3-(trifluoromethyl)phenyl)methanone (3o)

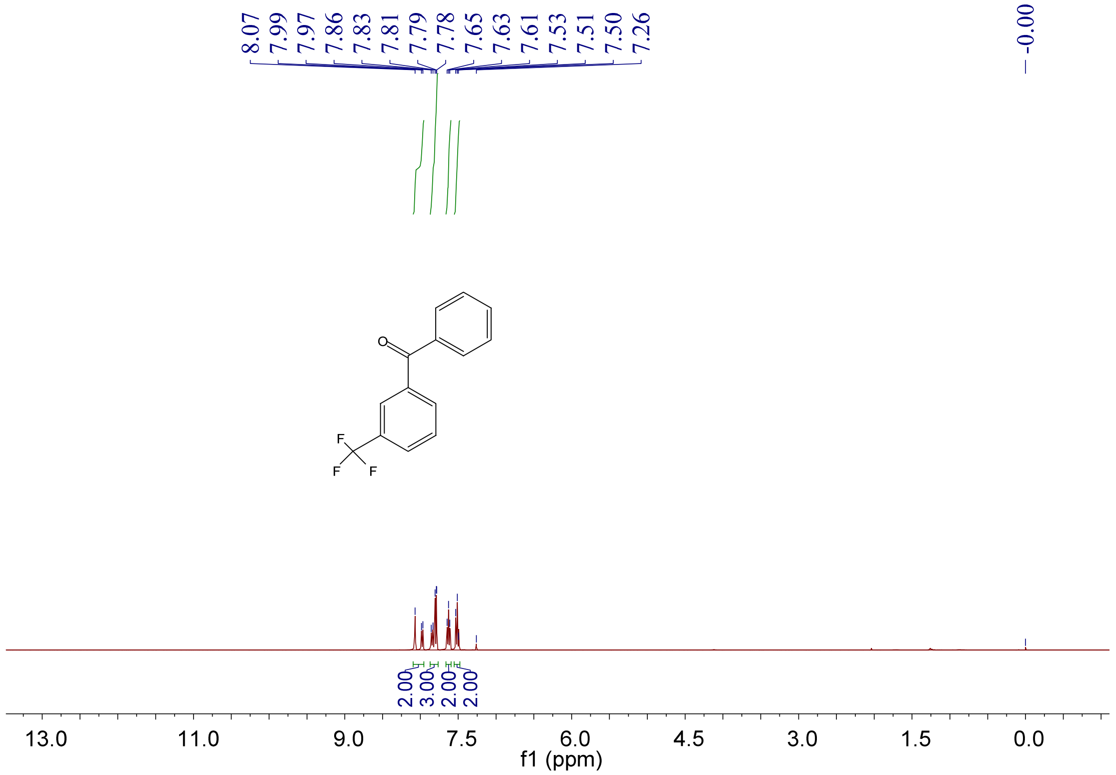

bis(4-chlorophenyl)methanone (3p)

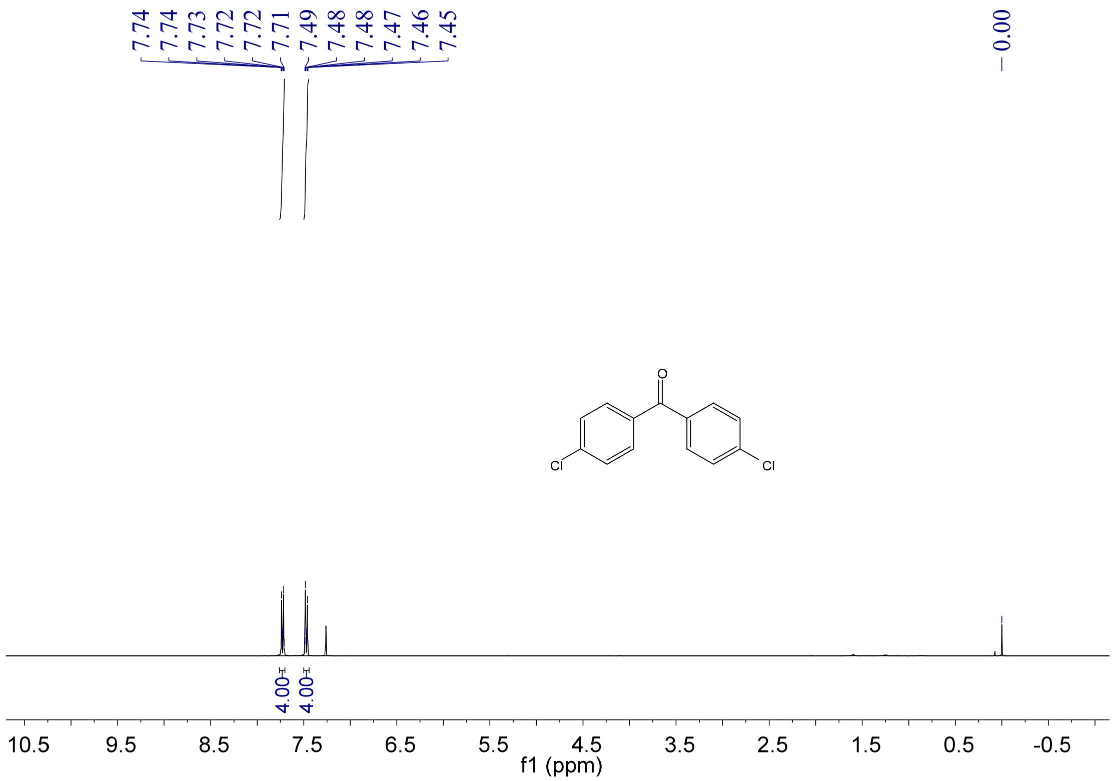

1-(naphthalen-2-yl)ethan-1-one (3q)

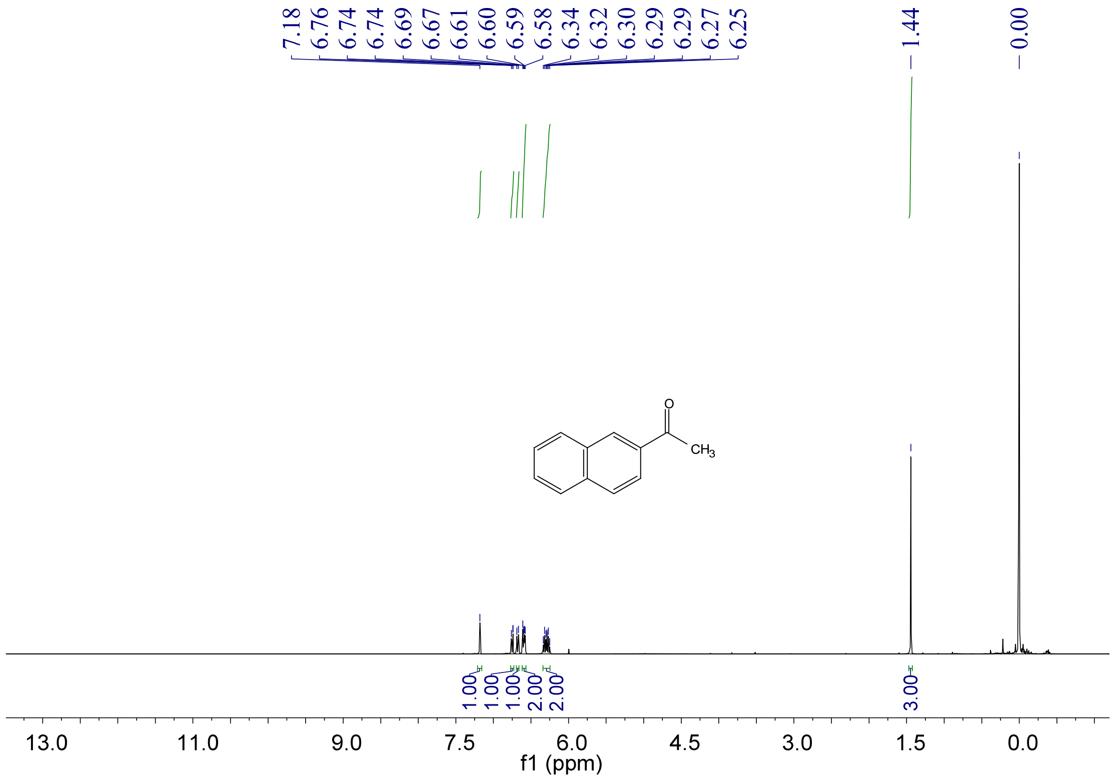

3,6-Di-tert-butyl-9H-fluoren-9-one(3r)

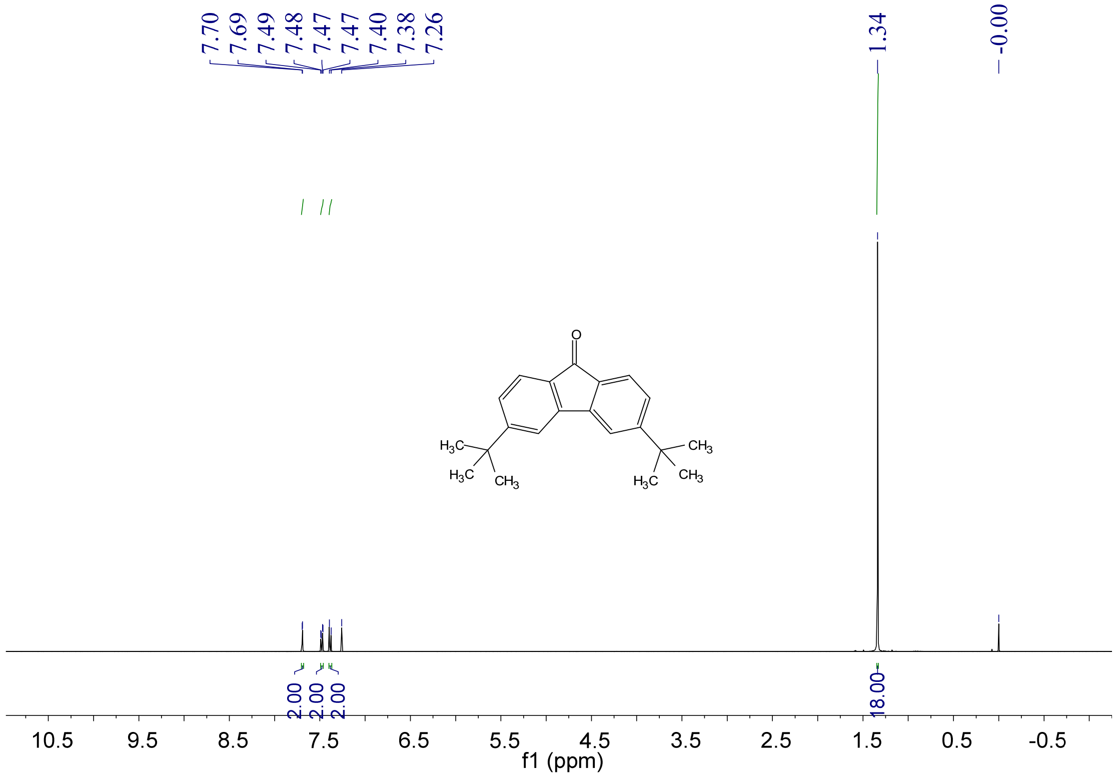

9H-xanthen-9-one (4b)

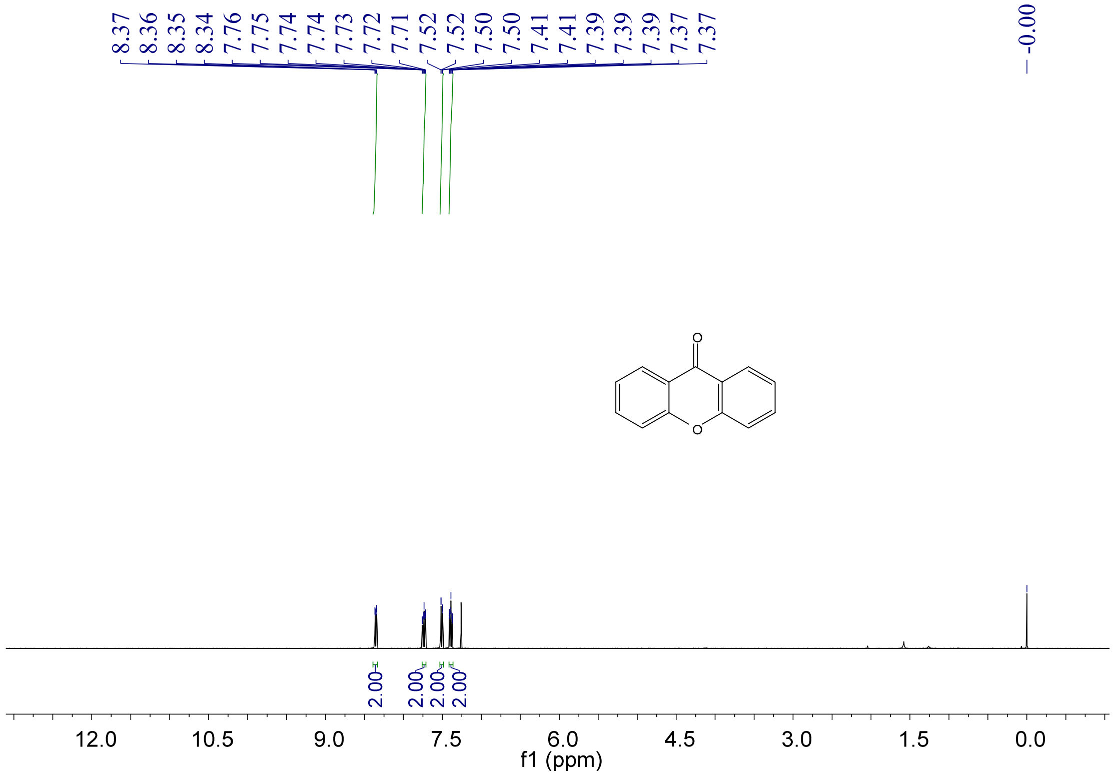

Supplement: Supplementary file 1 [file molecules-23-01883-s001.pdf]
